# Ginsenoside Rg1 antagonizes diabetic osteoporosis by regulating ferroptosis via mitochondrial membrane potential in H-type vascular endothelial cells

**DOI:** 10.3389/fragi.2026.1736263

**Published:** 2026-03-17

**Authors:** Mi Chen, Hongxiang Zheng, Rui Bai, Yingjie Huang, Guoyu Pang, Haixia Zhu, Zhuoxin Yi, Wenhui Chen

**Affiliations:** 1 School of Graduate, Guangxi University of Chinese Medicine, Nanning, China; 2 Faculty of Chinese Medicine Science, Guangxi University of Chinese Medicine, Nanning, China; 3 Department of Endocrinology, The First Affiliated Hospital of Guangxi University of Chinese Medicine, Nanning, China

**Keywords:** diabetic osteoporosis, ferroptosis, ginsenoside Rg1, H-type vascular endothelial cells, mitochondrial function

## Abstract

**Background:**

Endothelial dysfunction under high-glucose conditions is a key pathological process contributing to the development and progression of diabetic osteoporosis (DOP). High glucose-induced damage to H-type vascular endothelial cells (H-type ECs), including mitochondrial dysfunction, increased lipid peroxidation, and activation of ferroptosis, is considered a potential mechanism underlying bone loss and dysregulated bone metabolism in DOP. Diabetic osteoporosis is a common and severe complication in patients with diabetes, and current clinical treatment options remain limited. Ginsenoside Rg1 (Rg1), one of the main active components of ginseng, has been shown to possess antioxidant and anti-osteoporotic effects, but its underlying mechanisms in DOP remain unclear.

**Methods:**

In this study, spontaneously diabetic GK rats and high-glucose-treated H-type ECs were used to establish *in vivo* diabetic osteoporosis models and *in vitro* cell models, respectively. The effects of Rg1 on bone loss in GK rats as well as on mitochondrial function and lipid peroxidation in H-type ECs were evaluated. *In vivo* and *in vitro* experiments were conducted to investigate the potential mechanisms of Rg1 in regulating mitochondrial function and the SLC3A2/SLC7A11-GPX4 signaling pathway.

**Results:**

Our results showed that, compared with the model group, Rg1 at different doses effectively reduced systemic bone loss, with no significant difference between medium and high doses. Compared with the ferroptosis activator and inhibitor groups, Rg1 inhibited ferroptosis and promoted H-type vessel formation. Furthermore, *in vitro* experiments confirmed these findings and demonstrated that Rg1 activated the SLC3A2/SLC7A11-GPX4 signaling pathway, while modulating H-type ECs mitochondrial membrane potential, decreasing mitochondrial reactive oxygen species (mtROS), and increasing lipid peroxidation.

**Conclusion:**

Our study demonstrated that Rg1 promotes vessel–osteoblast coupling and regulates bone metabolism, thereby delaying the progression of diabetic osteoporosis (DOP). The underlying mechanism may involve activation of GPX4 expression in coordination with the regulation of H-type ECs mitochondrial membrane potential, leading to decreased mtROS levels and increased lipid peroxidation, ultimately intervening in ferroptosis. These findings highlight the GPX4–mitochondria cooperative regulation of H-type ECs ferroptosis by Rg1 and provide a new potential avenue for DOP therapy.

## 1 Introduction

Diabetic osteoporosis (DOP) is a skeletal complication of diabetes, emanating from persistently elevated blood glucose levels. Its defining features include diminished bone mass and impaired bone microarchitecture, which together elevate bone fragility and predispose individuals to fractures (Wang et al., 2025). Epidemiological data indicate that osteoporosis affects 20%–60% of the population with type 2 diabetes mellitus (T2DM). Notably, postmenopausal women with T2DM have a 1.7-fold increased susceptibility to hip fracture relative to their non-diabetic peers (Hofbauer et al., 2022). Current research on the pathogenesis of DOP predominantly focuses on dysregulated glucose metabolism and imbalanced bone metabolism, with limited investigation into its association with diabetic microvascular damage. Notably, T2DM patients often present with microvascular complications, and these are implicated in age-related bone loss, a phenomenon especially evident in cortical bone (Shanbhogue et al., 2017). The Haversian system, central to cortical bone remodeling, is highly dependent on vascular supply. A hallmark of H-type vessels, a specialized capillary subtype, is their capacity to coordinate osteogenesis with angiogenesis (Kusumbe et al., 2014). Therefore, targeting H-type vessels has emerged as a promising novel strategy for treating DOP.

Acting as central hubs in eukaryotic cells, mitochondria modulate iron, energy, and lipid metabolism. This regulatory function enables the stabilization of mitochondrial membrane potential (MMP) and lipid peroxide levels, thereby preserving mitochondrial-associated ferroptosis homeostasis (Battaglia et al., 2020). Cellular energy metabolism integrates three key processes: glycolysis, the tricarboxylic acid cycle, and the electron transport chain (ETC). A stable MMP is sustained primarily by the proton-pumping activity of the ETC (Di Lisa et al., 1995) Subsequently, the generated O_2_•^-^ is primarily dismutated into hydrogen peroxide (H_2_O_2_) (Robb et al., 2018). The Fenton reaction further processes H_2_O_2_ into highly reactive hydroxyl radicals (•OH), thereby initiating lipid peroxidation through radical-mediated attack on lipid molecules (Battaglia et al., 2020; Stockwell, 2022). Complexes I and III of the ETC serve as primary sites for mitochondrial reactive oxygen species (mtROS) generation, which occurs during the interaction of electrons (derived from NADH or FADH_2_) with molecular oxygen (Chouchani et al., 2016). Moreover, MMP hyperpolarization represents a significant stimulus for the generation of reactive oxygen species (ROS) (Korshunov et al., 1997). Interestingly, while serving as the main site for ROS generation, mitochondria are also subjected to significant oxidative damage from these same molecules (Marchi et al., 2012). The influx of ROS into mitochondria induced by iron overload disrupts MMP generation. This disruption promotes mitochondrial swelling and a further increase in ROS production, thereby establishing a self-perpetuating cycle (He et al., 2019). Moreover, ROS accumulation inhibits osteoblast (OB) activity (Zhang et al., 2017). It is therefore plausible that mitochondria-related ferroptosis is a key driver in the progression of osteoporosis.

In contrast, the term ferroptosis describes a unique mode of regulated cell death driven by dysregulated iron metabolism. Its core mechanism lies in iron-driven excessive ROS production and aberrant lipid peroxide accumulation, ultimately leading to alterations in mitochondrial membrane structure, peroxidative damage to cellular membrane phospholipids, and consequent cell death (Dixon et al., 2012). This cell death pathway is implicated in vascular endothelial cell (VEC) injury and the pathogenesis of osteoporosis. Studies have indicated that ferroptosis inducers can cause VECs to accumulate substantial ROS and exhibit typical ferroptotic characteristics (Xiao et al., 2019). Furthermore, studies in diabetic rat models have revealed pathologically elevated serum ferritin levels alongside a marked decrease in glutathione peroxidase 4 (GPX4) expression within bone tissue (Lin et al., 2022). The System Xc^−^/GSH/GPX4 axis is defined by its role as a master regulator of ferroptosis, executing this function through the enforcement of redox balance via its integrated antioxidant activity. Notably, the persistent hyperglycemia and metabolic disturbances in DOP elevate oxidative stress and impair the antioxidant system, particularly the GSH/GPX4 axis. Simultaneously, these disturbances affect the activation of osteoblasts and osteoclasts, further accelerating bone loss and the progression of osteoporosis (Bao et al., 2023). Thus, DOP represents a pathological condition intertwining diabetic microvascular complications and aberrant bone metabolism, in which dysregulation of the System Xc^−^/GSH/GPX4 axis is postulated to play a pivotal regulatory role.

As a key characteristic component, Ginsenoside Rg1 (Rg1) is abundant in Panax notoginseng and Panax ginseng. It has been demonstrated to not only promote angiogenesis and exert anti-osteoporotic effects (Jiang et al., 2024; Shi et al., 2009) but also to enhance antioxidant capacity, reduce lipid peroxidation and ROS levels, maintain GPX4 activity, and inhibit ferroptosis (Kong et al., 2024). We previously documented that Rg1 counters DOP progression in Goto–Kakizaki (GK) rats through its stimulatory effect on intraosseous H-type vessels and osteoblasts, a process governed by the Notch/Noggin/VEGF pathway that facilitates vascular-osteogenic coupling (Chen et al., 2022). However, whether and how Rg1 ameliorates DOP by regulating mitochondria-related ferroptosis via its antioxidant capacity remains elusive. Therefore, we hypothesize that Rg1 combats DOP by inhibiting ferroptosis in H-type vascular endothelial cells (H-type ECs) through the modulation of MMP, which in turn enhances vascular-osteogenic coupling. The primary aim of this investigation was to elucidate the molecular mechanisms through which Rg1 exerts its protective effects in DOP.

## 2 Materials and methods

### 2.1 Drugs and reagents

Detailed information on the batch numbers and manufacturers of the major drugs and reagents used in this study is provided in Supplementary Table S1.

### 2.2 Animals and experimental design

All animal procedures utilized ninety-five 6-month-old male GK rats and fifteen Wistar rats (200 ± 10 g), and were conducted in compliance with SPF standards. GK and Wistar rats were procured from different commercial suppliers: the former from Changzhou Cavens Experimental Animal Co., Ltd., and the latter from Changsha Tianqin Biotechnology Co., Ltd., with license numbers SCXK (Su) 2021-0013 and SCXK (Xiang) 2019-0013, respectively. Prior to experimentation, all rodents underwent a 14-day acclimatization period and were then housed in the Animal Experiment Center. Environmental parameters were strictly maintained at 22 °C ± 2 °C, 50% ± 5% relative humidity, with a 12/12 h light/dark cycle to ensure welfare and experimental stability. The research was performed under a protocol approved by the Institutional Animal Ethics Committee (Guangxi University of Chinese Medicine, Approval No. DW20240603-144). To establish the diabetic osteoporosis (DOP) model, five randomly selected GK and Wistar rats first underwent 7-day fasting blood glucose (FBG) monitoring. After confirming hyperglycemia (FBG ≥ 16.7 mmol/L), another five rats from each group were subjected to micro-CT scanning to verify successful model induction. Following confirmation of DOP, GK rats were randomized into four groups of ten. Treatment regimens consisted of saline for the MC group and Rg1 at 5, 10, or 20 mg/kg/day for the Rg1-L, Rg1-M, and Rg1-H groups, with all solutions administered via gavage. The remaining ten Wistar rats served as the Blank Control (BC) group and were given saline. After the 12-week regimen, an analysis of bone mineral density (BMD) by micro-CT served to guide the selection of the most effective Rg1 dose. For mechanistic insight, a separate cohort of fifty GK rats was allocated to five treatment groups (n = 10): Rg1 (10 mg/kg/day, i.g.), Ferrostatin-1 (0.655 mg/kg (Cheon et al., 2023), i.p., 3/week) (Molecular weight: 262.35 g/mol), RSL3 (100 mg/kg (Chen et al., 2025), i.p., 2/week), and the two corresponding combination therapy groups. Upon completion of this additional 12-week period, all animals were euthanized via 1% sodium pentobarbital anesthesia for the procurement of blood and bone samples.

#### 2.2.1 Micro-CT analysis for bone mineral density

The femurs were fixed and immersed in tissue fixative. Evaluation of bone microarchitecture was performed using a Skyscan 1176 high-resolution micro-CT system (Bruker microCT, Aartselaar, Belgium). Following reconstruction of 3D images in N-Recon software (v.X.X, Bruker microCT), a detailed morphometric analysis was undertaken using CT-Analyser (CTAN) software (v.X.X, Bruker microCT). The assessed microstructural parameters were: bone mineral density (BMD), bone volume fraction (BV/TV), trabecular number (Tb.N), trabecular thickness (Tb.Th), trabecular separation (Tb.Sp), and structure model index (SMI).

#### 2.2.2 Immunofluorescence (IF)

The immunofluorescence procedure was as follows. Deparaffinized and rehydrated sections were first treated with Proteinase K (37 °C, 8 min) for antigen retrieval and washed with PBS. Subsequently, endogenous peroxidases were inactivated by a 10-min incubation with 3% H_2_O_2_ in the dark. After blocking non-specific sites with goat serum (1 h), sections were incubated with primary antibodies against CD31 (1:200), Emcn (1:50), and Osterix (1:200) at 4 °C overnight. Following primary antibody incubation and PBS washes, a 1 h incubation in the dark with fluorescent secondary antibodies [FITC-conjugated goat anti-rabbit IgG (1:200), Cy5-conjugated goat anti-mouse IgG (1:200)] was performed. Nuclei were then labeled with DAPI, and signals for CD31, Emcn, and Osterix were captured by fluorescence microscopy.

#### 2.2.3 Measurement of malondialdehyde (MDA) content

MDA levels were assessed following a commercial kit’s instructions. The sample (0.1 mL) was placed in a microcentrifuge tube, to which 0.3 mL of the working solution was added. After vortex mixing and a 15-min incubation in a boiling water bath, the mixture was cooled and centrifuged. The MDA concentration in each sample was determined by measuring the absorbance at 532 nm after transferring 200 µL of the supernatant to a 96-well plate, with reference to a standard curve for quantification.

#### 2.2.4 Determination of glutathione (GSH) level

GSH levels were assayed using a commercial kit as per the manufacturer’s instructions. Briefly, tissue homogenates or cell lysates were prepared and processed as specified. The absorbance at 412 nm was determined for all wells with a microplate reader, prior to deriving the GSH concentration in each sample from a standard curve based on the GSH standards.

#### 2.2.5 Western blot analysis

Western blot was conducted using standard procedures. Briefly, proteins were extracted from samples using RIPA buffer supplemented with inhibitors. After centrifugation (12,000 × g, 20 min, 4 °C), the protein concentration in the supernatant was quantified with a BCA kit. Equal protein aliquots (20–30 μg) were resolved on 10% SDS-PAGE gels and transferred to PVDF membranes. After blocking with 5% non-fat milk/TBST (2 h), the membranes were incubated overnight at 4 °C with primary antibodies [SLC7A11 (1:1,000), SLC3A2 (1:1,000), GPX4 (1:1,000), GAPDH (1:5,000)]. Subsequent probing involved a 1-h incubation with HRP-conjugated secondary antibodies (1:1,000) at room temperature. After final washes, an enhanced chemiluminescence substrate was applied to generate signals from the protein bands, which were then imaged on a ChemiScope 5,300 Pro system (CLiNX, China). Quantitative analysis was carried out by measuring band density with ImageJ software.

#### 2.2.6 Quantitative reverse transcription polymerase chain reaction (RT-qPCR)

Quantification of gene expression was performed via quantitative real-time PCR (RT-qPCR). Following total RNA extraction from tissues or cells using TRIzol reagent, its concentration and purity were quantified by measuring A260/A280 ratios. cDNA was generated from equal RNA quantities via reverse transcription. Quantitative PCR was then conducted using a SYBR Green premix on a real-time detection system. Data analysis involved determining relative expression via the 2^−ΔΔCT^ method, normalizing all values to Gapdh. Table 1 contains the sequences of all primers used in this study.

**TABLE 1:** The primer sequences are used for RT-qPCR amplification.

Primer	Sequence
Rat-SLC7A11-F	TAT​GCT​GAA​TTG​GGT​ACG​AGC
Rat-SLC7A11-R	TAT​TAC​CAG​CAG​TTC​CAC​CCA
Rat-SLC3A2-F	CAC​TCC​CAA​CTA​TAA​GGG​CCA​GA
Rat-SLC3A2-R	ATT​CGC​CAG​CTT​TCC​CAC​AT
Rat-GPX4-F	CCA​TTC​CCG​AGC​CTT​TCA​AC
Rat-GPX4-R	GAA​CTC​GTG​GCT​GTT​GCT​G
Rat-GAPDH-F	TCT​CTG​CTC​CTC​CCT​GTT​CT
Rat-GAPDH-R	ATC​CGT​TCA​CAC​CGA​CCT​TC

#### 2.2.7 Hematoxylin and eosin (H&E) staining analysis

Bone tissues were fixed, decalcified, and embedded in paraffin. The paraffin sections were deparaffinized in xylene and rehydrated through a graded ethanol series, followed by hematoxylin staining, differentiation, and bluing. The sections were then counterstained with eosin, dehydrated, cleared, and mounted. Finally, the stained sections were observed and imaged under a light microscope.

### 2.3 Cell and experimental design

#### 2.3.1 Culture and identification of H-type vascular endothelial cells

Primary rat femoral head microvascular endothelial cells, a model system for *in vitro* angiogenesis, were commercially sourced from MirrorCell Biotechnology Co., Ltd. (product # RAT-iCell-s043, Shanghai, China). Cells were cultured in T75 flasks (5 × 10^5^ cells/flask) under standard conditions. Following expansion, experiments were conducted using passage three cells. The presence and identity of H-type ECs were verified via immunofluorescence staining for the characteristic markers Emcn and CD31.

#### 2.3.2 MTT assay

The MTT assay was employed to measure cell viability. Briefly, H-type ECs were cultured overnight in 96-well plates at 5 × 10^4^ cells per well. Following this, the culture medium was replaced with fresh medium containing Rg1 at specified concentrations (0, 10.3, 20.6, 41.2, 82.4, 164.8, and 200 μM), with consistent conditions across all groups. MTT treatment was performed at 24 and 48 h by adding 10 μL of solution to each well. This was followed by a 2 h incubation period. Thereafter, the formazan product was quantified by measuring the OD at 492 nm with a microplate reader.

#### 2.3.3 Cell treatment

Following a previously established method (Chen et al., 2022), H-type ECs were categorized into three groups differing only in medium composition: the CON group (5.5 mmol/L glucose) served as the control; the HG group was exposed to 32.8 mmol/L glucose; and the HG + Rg1 group received high glucose (32.8 mmol/L) along with 164.8 μmol/L Rg1.

#### 2.3.4 Detection of lipid peroxidation

Assessment of cellular lipid peroxidation was carried out via flow cytometry subsequent to BODIPY 581/591 C11 staining. Cells incubated with the probe for 1 h at 37 °C were harvested by centrifugation and washed. The oxidation-induced fluorescence shift was analyzed on a flow cytometer configured with a 488 nm laser for excitation, and emission filters for 530 nm (green, oxidized) and 585 nm (red, reduced) signals.

#### 2.3.5 Measurement of mitochondrial reactive oxygen species (mtROS) levels

Mitochondrial superoxide production was measured with MitoSOX™ Red. After harvesting and centrifugation, cells were stained with the probe (working solution prepared per instructions) for 30 min at 37 °C under dark conditions. Subsequently, cells were washed with PBS and nuclei were labeled with Hoechst 33,342. After final resuspension, cells were imaged using a fluorescence microscope (excitation/emission: 396/610 nm; Violet 610 channel).

#### 2.3.6 Cell transfection

For transfection, H-type ECs were first seeded in six-well plates (2 × 10^5^ cells/well) for 18 h. Cells were then transfected via the LipO6000™ reagent per the manufacturer’s protocol, employing siRNA against GPX4 or a scramble control. Following transfection, the medium was refreshed, and cells were maintained in culture for later experiments.

#### 2.3.7 Detection of mitochondrial membrane potential (MMP)

The JC-1 staining procedure was as follows: (1) prepare the working solution by diluting the 200× stock and mixing with 5× buffer; (2) harvest cells, resuspend in 0.5 mL working solution, and incubate at 37 °C for 20 min in the dark; (3) centrifuge and wash cells twice with staining buffer; (4) analyze immediately by flow cytometry. The ratio of red aggregated (585/590 nm) to green monomeric (514/529 nm) fluorescence intensity, as detected by fluorescence microscopy, served as an indicator of MMP.

### 2.4 Statistical analysis

The SPSS statistical package (v26.0) was employed for all data analysis. Normally distributed continuous data are presented as mean ± SD. For comparisons between two groups and among multiple groups, the Student’s t-test and one-way ANOVA were applied, respectively, followed by the LSD *post hoc* test when necessary. A p-value of less than 0.05 was considered statistically significant.

## 3 Results

### 3.1 Effects of Rg1 on bone microarchitecture in DOP rats

**FIGURE 1:**
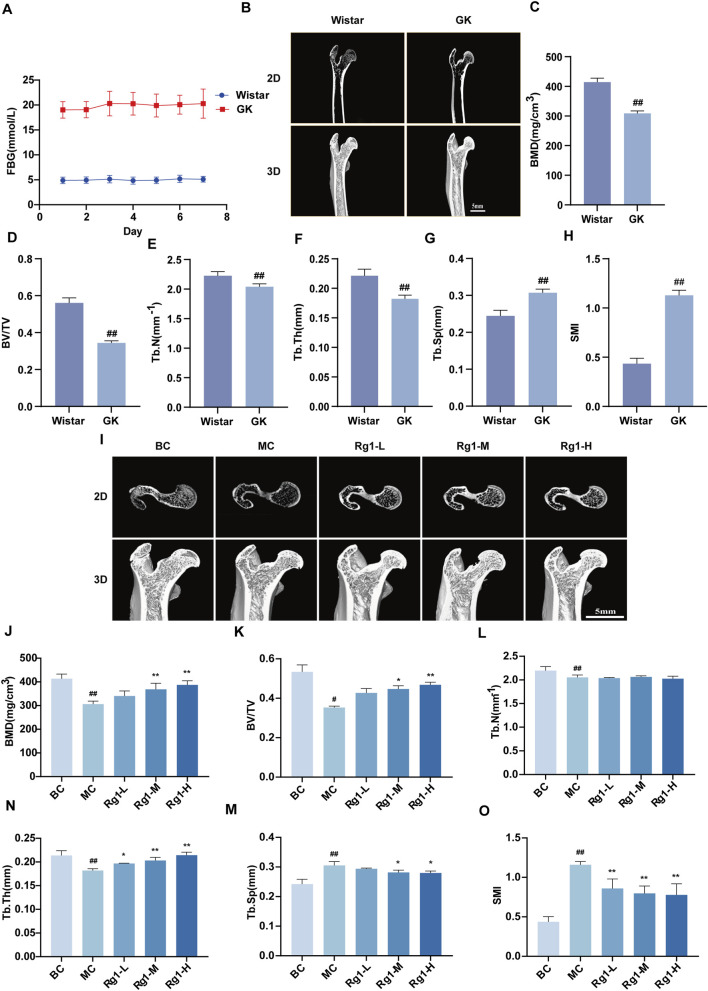
Effects of Rg1 on bone microstructure in DOP rats. **(A)** Fasting blood glucose levels in Wistar and GK rats for 7 consecutive days. **(B)** Two-dimensional and three-dimensional micro-CT images of femurs from Wistar and GK rats. **(C–H)** Quantitative analysis of BMD **(C)**, BV/TV **(D)**, Tb.N **(E)**, Tb.Th **(F)**, Tb.Sp **(G)**, and SMI **(H)** in femurs of Wistar and GK rats after 2 weeks of adaptive feeding. **(I)** Two-dimensional and three-dimensional micro-CT images of femurs from rats in each group after 12 weeks of treatment. **(J–O)** Quantitative analysis of bone microstructure in femurs of rats from each group after 12 weeks of treatment. Data are presented as mean ± SD. #P < 0.05 and ##P < 0.01 vs. Wistar/BC, *P < 0.05 and **P < 0.01 vs. MC.

Continuous blood glucose monitoring for 7 days revealed that GK rats exhibited hyperglycemic levels (Figure 1A). Micro-CT analysis of femoral bones showed that trabecular bone in Wistar rats displayed a dense, interconnected, and intact porous network structure. Conversely, GK rats displayed a loss of structural integrity in bone, manifesting as reduced bone mass, compromised microarchitecture, thinner trabeculae, and enlarged marrow spaces. Successful induction of the DOP model was confirmed by micro-CT analysis, which demonstrated a significant decrease in BMD, BV/TV, Tb.N, and Tb.Th, coupled with an increase in Tb.Sp and SMI (Figures 1B–H). After Rg1 treatment, bone loss was markedly alleviated (Figures 1I–O), indicating that Rg1 mitigated DOP-induced bone loss. H&E staining revealed that, in the model group, trabecular bone was markedly reduced in number, became thinner and discontinuous with evident fragmentation, and exhibited a disorganized architecture and impaired structural integrity; cortical bone thickness was decreased; and the bone marrow cavity was enlarged with increased adipocyte infiltration. In contrast, Rg1 dose-dependently ameliorated these pathological alterations in bone tissue (Supplementary Figure S1). Notably, the Rg1-M and Rg1-H groups yielded comparable effects on bone microarchitecture, with no statistically significant differences observed. Based on these findings, the medium dose of Rg1 was selected for subsequent *in vivo* experiments to further investigate its role in regulating ferroptosis during DOP progression.

### 3.2 Effects of Rg1 on osteoblasts and H-type vessels

**FIGURE 2:**
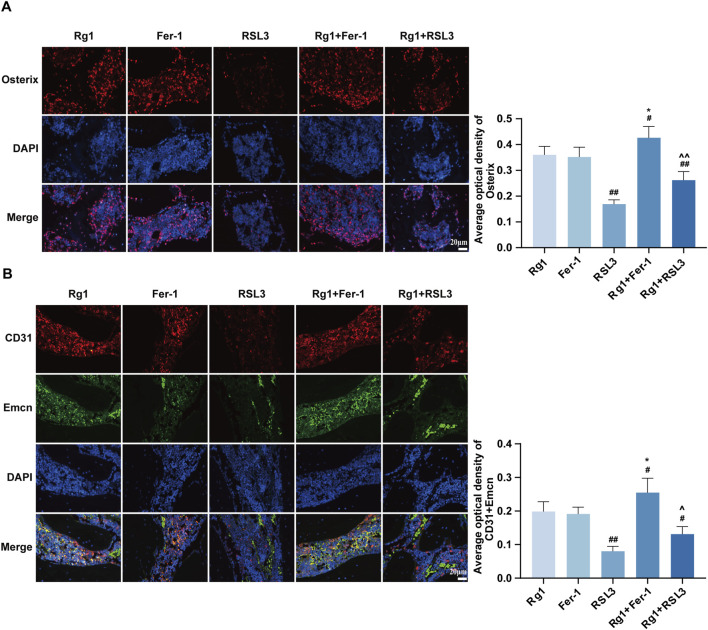
Effects of Rg1 on osteoblasts and H-type blood vessels. **(A)** Immunofluorescence staining images of Osterix in bone tissues of rats from each group and quantitative analysis of the mean relative level of Osterix. **(B)** Immunofluorescence staining images of Emcn and CD31 in bone tissues of GK rats from each group and quantitative analysis of the mean relative levels of Emcn and CD31. Data are presented as mean ± SD. #P < 0.05 and ##P < 0.01 vs. Rg1, *P < 0.05 vs. Fer-1, ^∧^P < 0.05 and ^∧∧^P < 0.01 vs. RSL3.

To delineate the role of ferroptosis in DOP, we utilized pharmacological modulators: Ferrostatin-1 (Fer-1) as an inhibitor and RSL3 as an activator, treating rats accordingly. The expression of the osteogenic marker Osterix was examined to determine whether Rg1 promotes osteoblast formation through modulation of ferroptosis. Immunofluorescence (Figure 2A) demonstrated similar efficacy between the Rg1 and Fer-1 groups in promoting osteoblast formation, whereas Rg1 alone was sufficient to reverse the inhibitory action of RSL3. As a highly vascularized tissue, bone is critically dependent on its vasculature for metabolic homeostasis, repair, and regeneration. The H-type vessel subpopulation can be identified among capillaries by its notable co-expression of the surface markers CD31 and Emcn (Kusumbe et al., 2014). Immunofluorescence staining demonstrated that RSL3 suppressed CD31 and Emcn expression, whereas Rg1 partially reversed this inhibitory effect on H-type vessel formation (Figure 2B).

### 3.3 Effects of Rg1 on ferroptosis in DOP rats

**FIGURE 3:**
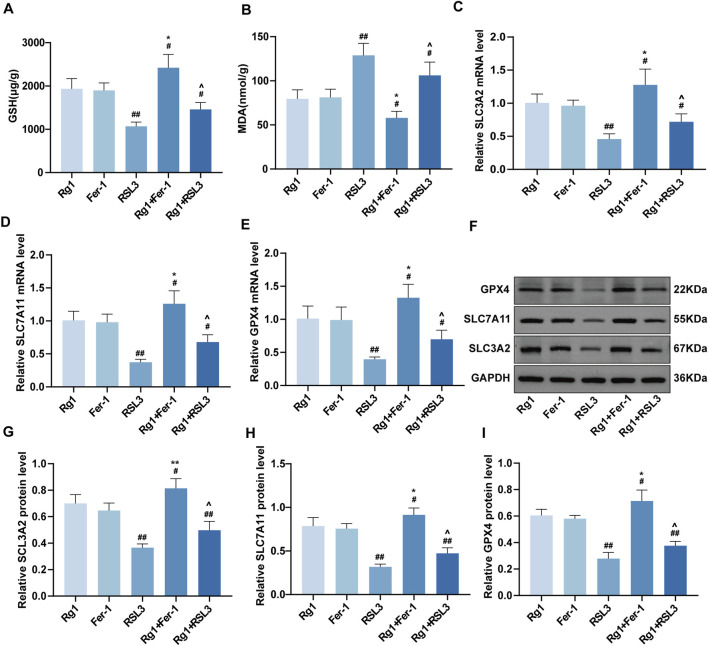
Effects of Rg1 on ferroptosis in DOP rats. **(A)** Analysis of GSH content in bone tissue. **(B)** Analysis of MDA content in bone tissue. **(C–E)** The mRNA expression levels of SLC3A2, SLC7A11, and GPX4 in rat bone tissues from each group were detected by RT-qPCR. **(F)** Representative Western blot images showing the protein bands of SLC3A2, SLC7A11, and GPX4 in bone tissues from each group. **(G–I)** Quantitative analysis of SLC3A2, SLC7A11, and GPX4 protein expression levels in bone tissues. Data are presented as mean ± SD. #P < 0.05 and ##P < 0.01 vs. Rg1, *P < 0.05 and **P < 0.01 vs. Fer-1, ^∧^P < 0.05 vs. RSL3.

During ferroptosis, GSH levels typically decrease while MDA levels increase, serving as key indicators of lipid peroxidation. Analysis of bone tissue revealed that Rg1 and Fer-1 showed no significant difference in GSH levels (Figure 3A), but Rg1 significantly reduced MDA levels (Figure 3B), indicating attenuation of oxidative stress and ferroptosis in DOP rats. To delineate Rg1’s role in ferroptosis regulation, we examined the SLC3A2/SLC7A11-GPX4 pathway, measuring the expression of its corresponding mRNAs and proteins. Results showed that Rg1 and Fer-1 groups exhibited comparable expression of SLC3A2, SLC7A11, and GPX4 mRNA, while Rg1 reversed RSL3-induced downregulation of these genes (Figures 3C–E). Western blot results further validated the RT-qPCR findings (Figures 3F–I). Collectively, these results indicate that Rg1, similar to Fer-1, restores antioxidant capacity, reduces lipid peroxidation, and suppresses ferroptosis in DOP rats.

### 3.4 Determination of optimal Rg1 concentration for H-type ECs intervention

**FIGURE 4:**
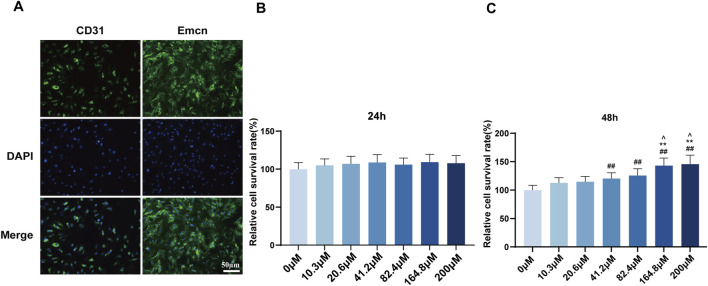
Screening of the optimal concentration of Rg1 for intervening in H-type ECs. **(A)** Immunofluorescence detection of Emcn and CD31 expression levels. **(B,C)** Cell viability rates under different concentrations of Rg1 were detected by MTT assay at 24 h and 48 h, respectively. ##P < 0.01 vs. 0µM, **P < 0.01 vs. 41.2µM, ^∧^P < 0.05 vs. 82.4 µM.

To elucidate the mechanism between H-type ECs and ferroptosis, primary femoral head microvascular endothelial cells were cultured to passage three and characterized by immunofluorescence. Consistent with previous findings (Jin et al., 2024), strong positive expression of Emcn and CD31 confirmed the H-type ECs identity (Figure 4A). Cell viability was determined following treatment with different concentrations of Rg1. At 24 h, MTT assay indicated no inter-group differences in viability (Figure 4B). By 48 h, concentration-dependent effects became apparent from 41.2 μmol/L upwards, and viability plateaued at concentrations exceeding 164.8 μmol/L (Figure 4C). Thus, 164.8 μmol/L Rg1 for 48 h was selected for subsequent experiments.

### 3.5 Rg1 inhibits ferroptosis in H-type ECs

**FIGURE 5:**
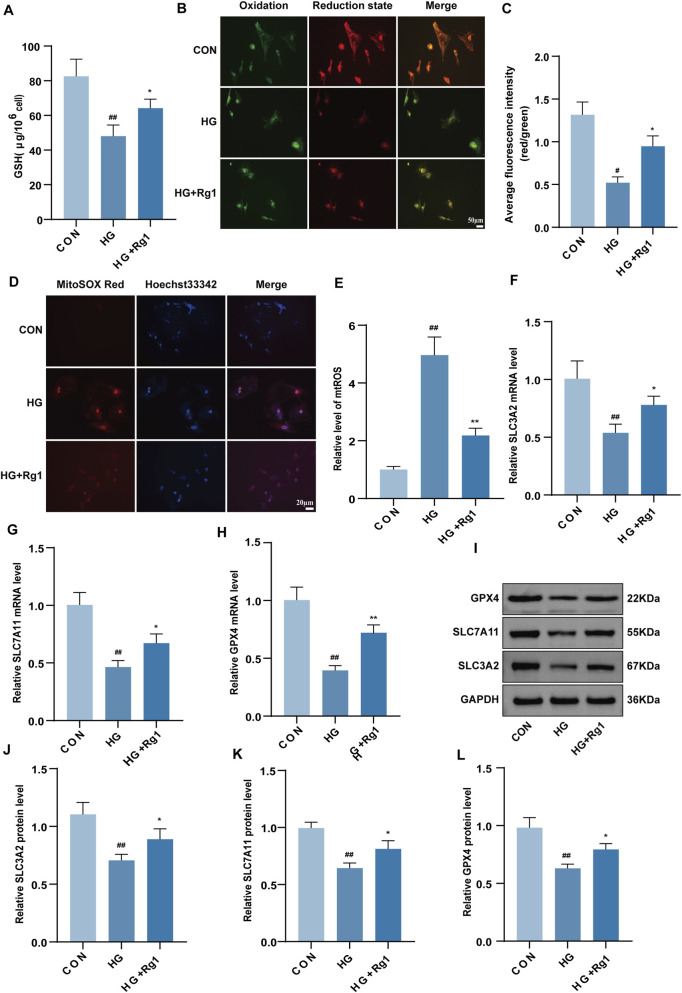
Rg1 inhibits ferroptosis in H-type ECs. **(A)** Analysis of GSH content in H-type ECs from each group after treatment. **(B,C)** Fluorescent probe detection images of lipid peroxidation levels in H-type ECs from each group and their quantitative analysis. **(D,E)** Fluorescent probe detection images of mtROS levels in H-type ECs from each group and their quantitative analysis. **(F–H)** RT-qPCR detection of SLC3A2 **(F)**, SLC7A11 **(G)**, and GPX4 **(H)** mRNA expression levels in H-type ECs from each group. **(I)** Western blot detection of SLC3A2, SLC7A11, and GPX4 protein band expression images in H-type ECs from each group. **(J–L)** Quantitative analysis of SLC3A2 **(J)**, SLC7A11 **(K)**, and GPX4 **(L)** protein expression levels in H-type ECs. Data are expressed as mean ± SD. ##P < 0.01 vs. CON, *P < 0.05 and **P < 0.01 vs. HG.

To further evaluate whether Rg1 suppresses ferroptosis in H-type ECs, GSH and lipid peroxidation were examined. Rg1 restored the decreased GSH levels induced by high glucose (Figure 5A). Similarly, lipid peroxidation levels followed the same trend (Figures 5B,C), confirming that Rg1 alleviated oxidative damage. mtROS fluorescence analysis showed that high glucose significantly increased mtROS levels, whereas Rg1 reversed this effect (Figures 5D,E). In light of the critical function of the SLC3A2/SLC7A11-GPX4 axis in ferroptosis (Hu et al., 2024), Rg1 was found to abrogate the high glucose-induced mRNA downregulation of SLC7A11, SLC3A2, and GPX4, as evidenced by RT-qPCR (Figures 5F–H). Western blot data demonstrated a reversal by Rg1 of the suppressed protein expression of these targets (Figures 5I–L). Collectively, Rg1 upregulated SLC7A11, SLC3A2, and GPX4 expression, leading to attenuated lipid peroxidation and mitochondrial ROS accumulation, which ultimately suppressed ferroptosis in H-type ECs.

### 3.6 Rg1 modulates mitochondrial membrane potential to attenuate high glucose–induced ferroptosis in H-type ECs

**FIGURE 6:**
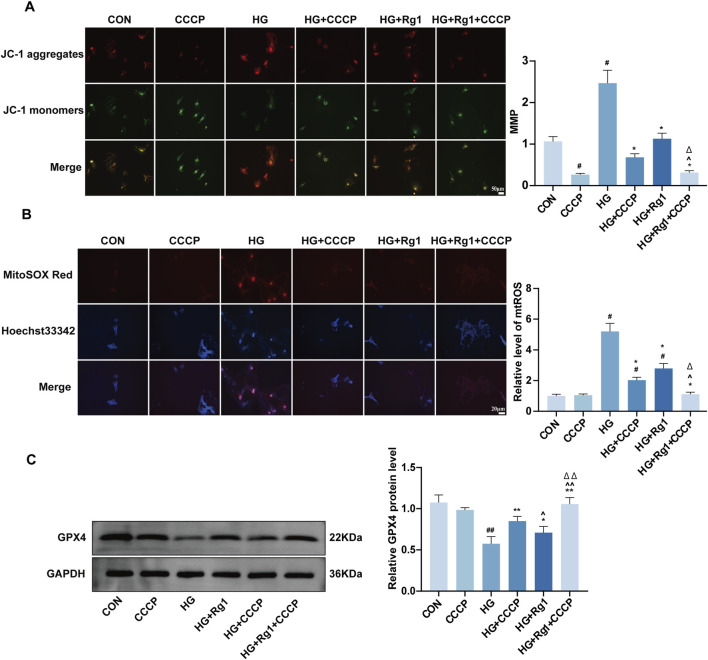
Rg1 intervenes in high glucose-induced ferroptosis in H-type ECs by regulating mitochondrial membrane potential. **(A)** Fluorescent probe detection images of mitochondrial membrane potential in H-type ECs from each group and their quantitative analysis. **(B)** Quantitative analysis of mtROS expression levels in H-type ECs from each group and their quantitative analysis. **(C)** GPX4 protein band expression images in H-type ECs from each group and their quantitative analysis. Data are expressed as mean ± SD. #P < 0.05 and ##P < 0.01 vs. CON, *P < 0.05 and **P < 0.01 vs. HG, ^∧^P < 0.05 and ^∧∧^P < 0.01 vs. HG + CCCP, △P < 0.05 and △△P < 0.01 vs. HG + Rg1.

Accumulation of mtROS is closely associated with MMP(DeHart et al., 2018) and ferroptosis (Song et al., 2024), while GPX4 acts as a critical regulator of this process (Cui et al., 2022). We hypothesized that Rg1 might modulate mtROS levels and GPX4 expression by regulating MMP. Treatment with the mitochondrial uncoupler CCCP confirmed this hypothesis. CCCP treatment increased MMP levels in HG-treated cells (P < 0.05), whereas both Rg1 and CCCP alone significantly reduced MMP (Figure 6A). It should be noted that under combined high-glucose and CCCP treatment, the MMP remained higher than that observed with CCCP treatment alone. This phenomenon may be attributable, at least in part, to the presence of 10% fetal bovine serum (Soutar et al., 2019) in the culture medium, as serum proteins can partially bind CCCP and thereby attenuate its depolarizing effect. In addition, high glucose enhances electron transport chain–driven proton pumping, which may partially compensate for CCCP-mediated proton leak and establish a new dynamic equilibrium of the proton gradient. Notably, combined treatment with Rg1 and CCCP exerted a stronger effect, consistent with mtROS fluorescence results (Figure 6B). Moreover, CCCP enhanced GPX4 protein expression, and Rg1 treatment similarly increased GPX4 levels (Figure 6C). These findings suggest that Rg1 may inhibit ferroptosis in H-type ECs by suppressing MMP and mtROS accumulation while upregulating GPX4 expression.

### 3.7 Rg1 regulates GPX4 to mitigate high glucose–induced ferroptosis in H-type ECs

**FIGURE 7:**
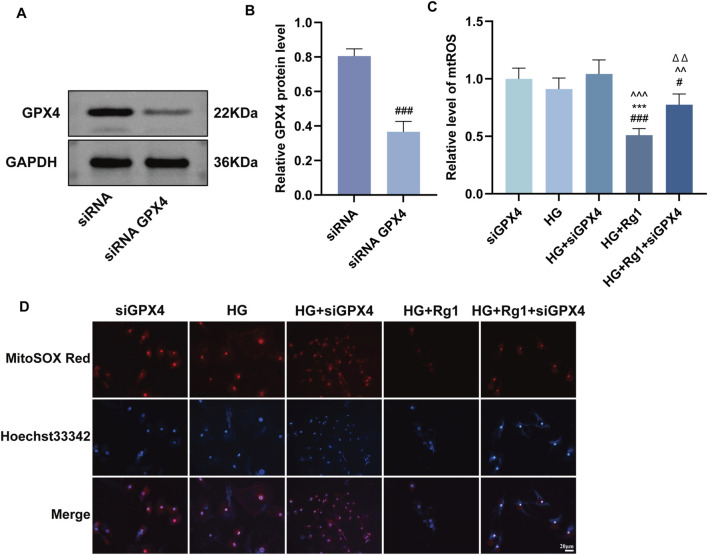
Rg1 regulates GPX4 to intervene in high glucose-induced ferroptosis in H-type ECs. **(A,B)** GPX4 protein band images and their quantitative analysis after transfection of GPX4 gene-silencing plasmids into H-type ECs. **(C,D)** Fluorescent probe detection images of mtROS expression levels in H-type ECs from each group and their quantitative analysis. Data are expressed as mean ± SD. #P < 0.05 and ##P < 0.01 vs. siRNA/siGPX4, **P < 0.01 vs. HG, ^∧∧^P < 0.01 vs. HG + siGPX4, △△P < 0.01 vs. HG + Rg1.

Inhibition of GPX4 activity accelerates ferroptosis (Doll et al., 2017). To determine whether Rg1 suppresses ferroptosis via the GPX4 pathway, siRNA-mediated GPX4 knockdown was performed in H-type ECs. Western blot analysis confirmed successful transfection, as GPX4 protein expression was markedly reduced in the si-GPX4 group compared with siRNA controls (Figures 7A,B). Furthermore, compared with the HG or HG + siGPX4 groups, Rg1 treatment significantly reduced mtROS levels (Figures 7C,D). These results indicate that even under GPX4 knockdown conditions, Rg1 attenuates mtROS production and mitigates ferroptosis in H-type ECs.

## 4 Discussion

Bone is highly vascularized, and its vascular networks not only provide nutritional support but also serve as a structural framework regulating dynamic bone remodeling. The coupling of vascularization and bone formation is essential for successful bone defect repair. Upon injury, blood vessels migrate to the defect site, supplying nutrients, oxygen, and minerals essential for bone matrix formation and mineralization (Zhang et al., 2020). H-type vessels, marked by high CD31 and Emcn expression, maintain bone homeostasis and fracture resistance by secreting VEGF and other factors to drive osteogenic differentiation in MSCs(Ramasamy et al., 2014). Previous studies reported that type I diabetic mice exhibited impaired H-type vessels, leading to uncoupled angiogenesis and osteogenesis and suppressed bone formation (Hu et al., 2021). Our previous work also confirmed that DOP rats displayed significantly reduced H-type vessels and decreased bone mineral density, indicating disrupted coupling between angiogenesis and osteogenesis (Jin et al., 2024), while Rg1 treatment reversed these changes (Chen et al., 2022). Consistent with these findings, the current study further demonstrates that Rg1 enhances H-type ECs expression in DOP rat femora. Moreover, Rg1 has been shown to downregulate MMP and suppress ROS accumulation (Liu et al., 2022). Taken together, these results indicate that ferroptosis within H-type ECs is a key driver of DOP progression, a pathogenic mechanism that Rg1 counteracts through the regulation of associated ferroptotic pathways.

While Rg1, a ginseng-derived compound, is known to modulate ferroptosis via multiple pathways (Kong et al., 2024; Tan et al., 2024), its specific mechanism in DOP remains to be fully defined. Since the execution of ferroptosis is ultimately characterized by aberrant lipid peroxidation (Liang et al., 2022), our investigation centered on the central pathway governing this process. The selenoenzyme GPX4 plays a central role by catalyzing the reduction of lipid peroxides to non-toxic lipid alcohols, thus preventing peroxidation propagation and suppressing ferroptosis (Ma et al., 2022; Seibt et al., 2019). This function is critically supported by the SLC3A2/SLC7A11-GPX4 pathway (Hu et al., 2024). Upstream, the uptake of cystine—a crucial precursor for glutathione (GSH) synthesis—is mediated by System Xc–. This transporter is a heterodimer comprising the SLC7A11 and SLC3A2 subunits. This process sustains the cellular redox balance and limits lipid peroxide accumulation, thereby conferring protection against oxidative damage (Dixon et al., 2012; Pang et al., 2025). Our data demonstrate that Rg1 upregulates both mRNA and protein expression of SLC3A2, SLC7A11, and GPX4 within this pathway, leading to a significant attenuation of lipid peroxidation, a finding corroborated by our *in vitro* experiments. Notably, Rg1 exhibited a slightly stronger overall protective trend in certain parameters, which may be attributable to its distinct mechanism of action compared with Fer-1. Fer-1 primarily functions as a lipid radical scavenger at the downstream stage of the ferroptosis pathway, whereas Rg1 may exert multi-target regulatory effects at more upstream levels by modulating mitochondrial function, suppressing reactive oxygen species generation, and enhancing endogenous antioxidant defense systems, thereby conferring broader and more sustained cytoprotective effects.

Mitochondria, core cellular organelles, are fundamental to metabolic and redox balance. Their dysfunction under disease conditions can disrupt skeletal homeostasis by impairing the osteoblast-osteoclast equilibrium (Chen et al., 2021; Langdahl et al., 2017). A key feature of functional decline is altered MMP (Tatarczuch et al., 2025). Hyperpolarization creates a state of heightened inner membrane potential, which drives excessive ROS generation via accelerated redox reactions (Shin and Cho, 2025). This mitochondrially derived oxidative stress is a critical conduit to ferroptosis. Indeed, inducers such as Erastin and RSL3 trigger a vicious cycle by inhibiting System Xc−/GPX4, leading to GSH depletion, ETC impairment, MMP perturbation, and sustained ROS accumulation that executes ferroptosis (Gao et al., 2019). The efficacy of MitoQ in countering GPX4 deficiency-induced ferroptosis highlights the central role of mitochondrial ROS (Jelinek et al., 2018). Grounded in this rationale, we hypothesized that Rg1 confers protection by modulating MMP. Experimental inhibition of MMP with CCCP in high glucose-treated H-type ECs revealed that elevated MMP, mtROS buildup, and suppressed GPX4 were interconnected pathologies. CCCP partially reversed this triad, and the addition of Rg1 enhanced the therapeutic outcome by synergistically lowering mtROS and upregulating GPX4, thereby preventing cell death.

RSL3, as a ferroptosis inducer, primarily triggers ferroptosis by directly inhibiting GPX4 activity. In our *in vivo* experiments, we similarly observed that RSL3 inhibited GPX4 activity in DOP rats, thereby accelerating the ferroptosis process. The suppressive effect of RSL3 on GPX4 was partially alleviated by Rg1 co-treatment, underscoring the central role of GPX4 in combating ferroptosis, as it has been reported to protect against cell death triggered by 12 distinct inducers (Yang et al., 2014). Therefore, in subsequent experiments, we chose to silence the GPX4 gene to observe the effect of Rg1 on DOP. This study found that after silencing the GPX4 gene, Rg1 could still restore the elevated mtROS levels.

Although we identified that Rg1 inhibits ferroptosis and alleviates DOP by targeting MMP and GPX4 in H-type ECs, the functional interplay between MMP and GPX4 was not definitively established. However, the present study still has several limitations. First, although Rg1 was shown to simultaneously regulate MMP and GPX4 expression, accompanied by reduced mtROS levels and alleviated ferroptotic features, the current evidence mainly supports their parallel changes under Rg1 intervention and is insufficient to establish that alterations in MMP constitute an upstream causal event for GPX4 regulation. Under high-glucose conditions, H-type ECs exhibited elevated MMP, increased mtROS accumulation, and reduced GPX4 expression, all of which were reversed by Rg1 treatment. In contrast, although the mitochondrial uncoupler CCCP reduced MMP and mtROS levels, it did not induce significant changes in GPX4 protein expression under our experimental conditions. Moreover, Rg1 remained effective in significantly reducing high glucose–induced mtROS accumulation even when GPX4 was silenced. These findings suggest that Rg1 may regulate mitochondrial functional status and the GPX4 antioxidant system in a parallel or coordinated manner; however, their direct causal relationship remains to be further clarified. Future studies employing specific manipulation of MMP, modulation of mitochondrial status under different GPX4 expression backgrounds, and the use of mitochondrial ROS scavengers are warranted to further dissect the hierarchical relationship among MMP, mtROS, and GPX4. Second, the observation that Rg1 markedly reduced mtROS levels even under GPX4 knockdown conditions indicates that its mitochondrial protective effects are not entirely dependent on the GPX4 antioxidant axis and may involve multiple regulatory mechanisms, the precise molecular pathways of which remain to be elucidated. A reasonable hypothesis is that Rg1 may reduce electron leakage and ROS generation by modulating MMP and ETC activity, activate alternative antioxidant defense pathways such as the Nrf2-mediated response and mitochondrial antioxidant systems (including SOD2, PRDX3/5, and the mitochondrial glutathione system), or improve mitochondrial structural and functional integrity to enhance resistance to oxidative stress. Further studies assessing ETC complex activity, Nrf2 signaling, and mitochondrial antioxidant enzyme expression, as well as employing ETC inhibitors or mitochondria-targeted antioxidants for functional blockade, are required to validate these potential mechanisms. Finally, the present study primarily focused on mitochondrial functional parameters, such as MMP and mtROS, and did not include direct assessment of mitophagy, which limits a comprehensive understanding of mitochondrial quality control mechanisms. Given the crucial role of mitophagy in clearing damaged mitochondria and maintaining cellular homeostasis, further research is warranted to clarify whether Rg1 exerts its mitochondrial protective and anti-ferroptosis effects at least partially through regulating mitophagy.

## 5 Conclusion

**FIGURE 8:**
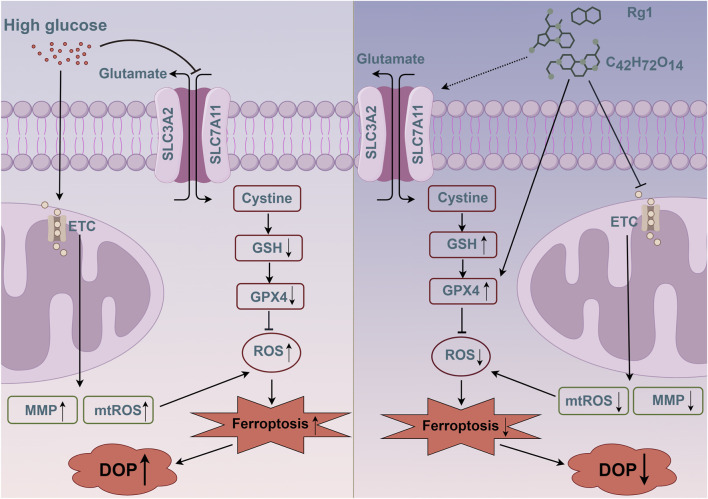
Schematic diagram of the pathway by which Rg1 regulates MMP and GPX4 in H-type ECs to trigger ferroptosis, thereby affecting DOP. Rg1 intervention may alleviate ferroptosis-mediated DOP progression by reducing MMP in H-type ECs, improving mtROS accumulation, and promoting the activation of the SLC3A2/SLC7A11-GPX4 pathway.

In conclusion, our findings illuminate a novel mechanism whereby Rg1 counteracts diabetic osteoporosis by suppressing ferroptosis via modulation of MMP and enhancement of GPX4 expression (Figure 8). These results highlight the pivotal role of ferroptosis in DOP and the multi-targeting nature of Rg1, which concurrently promotes osteogenic and H-type vascular coupling. The precise pathways governing Rg1’s control over mitochondrial membrane potential remain a subject for future investigation.

## Data Availability

The original contributions presented in the study are included in the article/Supplementary Material, further inquiries can be directed to the corresponding author.
