# Supplementary material for: Ginsenoside Rg1 antagonizes diabetic osteoporosis by regulating ferroptosis via mitochondrial membrane potential in H-type vascular endothelial cells
Source: Front Aging. 2026 Mar 17;7:1736263. doi: 10.3389/fragi.2026.1736263 (PMC13036096; doi:10.3389/fragi.2026.1736263)
Supplement: Supplementary file 1 [file Table1.docx]

**Supplementary Table 1.** List of Major Drugs and Reagents

| Batch number | manufacturer | Description |
| --- | --- | --- |
| S33043 | Shanghai Yuanye Bio-Technology Co | Ginsenoside Rg1 |
| HY-100579 | MedChemExpress | Ferrostatin-1 |
| HY-100218A | MedChemExpress | RSL3 |
| HY-00941 | MedChemExpress | CCCP |
| GB22303 | Servicebio | FITC-conjugated Goat Anti-Rabbit IgG |
| GB27301 | Servicebio | Cy5-conjugated Goat Anti-Mouse IgG |
| GB111900 | Servicebio | Anti-SP7/Osterix Rabbit Polyclonal Antibody |
| GB120005 | Servicebio | Anti-CD31 Mouse Monoclonal Antibody |
| GB112648 | Servicebio | Anti-Emcn Rabbit Polyclonal Antibody |
| BC1175 | Beijing Solarbio Science & Technology Co | Reduced Glutathione (GSH) Assay Kit |
| BC0025 | Beijing Solarbio Science & Technology Co | Malondialdehyde (MDA) Assay Kit |
| M1020 | Beijing Solarbio Science & Technology Co | MTT Cell Proliferation and Cytotoxicity Assay Kit |
| PAB48941 | Bioswamp | GPX4 Polyclonal Antibody |
| PAB38415 | Bioswamp | SLC7A11/xCT Polyclonal Antibody |
| iCell-s043-002r | iCell Bioscience Inc | Microvascular Endothelial Cell Medium |
| DF7468 | Affinity Biosciences | SLC3A2 Antibody |
| AF6191 | Affinity Biosciences | CD31 Antibody |
| DF13357 | Affinity Biosciences | Anti-Emcn Rabbit Polyclonal Antibody |
| S0061S | Beyotime Biotechnology | MitoSOX™ Red |
| S0043S | Beyotime Biotechnology | BODIPY® C11 |
| C2006 | Beyotime Biotechnology | JC-1 MMP kit |
| C0526 | Beyotime Biotechnology | LiPo6000™ transfection reagent |
| G8003-100ML | Servicebio | Fetal bovine serum (FBS) |
| GB23303 | Servicebio | HRP-conjugated goat anti-rabbit |
