# Supplementary figures and images for: Ginsenoside Rg1 antagonizes diabetic osteoporosis by regulating ferroptosis via mitochondrial membrane potential in H-type vascular endothelial cells
Source: Front Aging. 2026 Mar 17;7:1736263. doi: 10.3389/fragi.2026.1736263 (PMC13036096; doi:10.3389/fragi.2026.1736263)

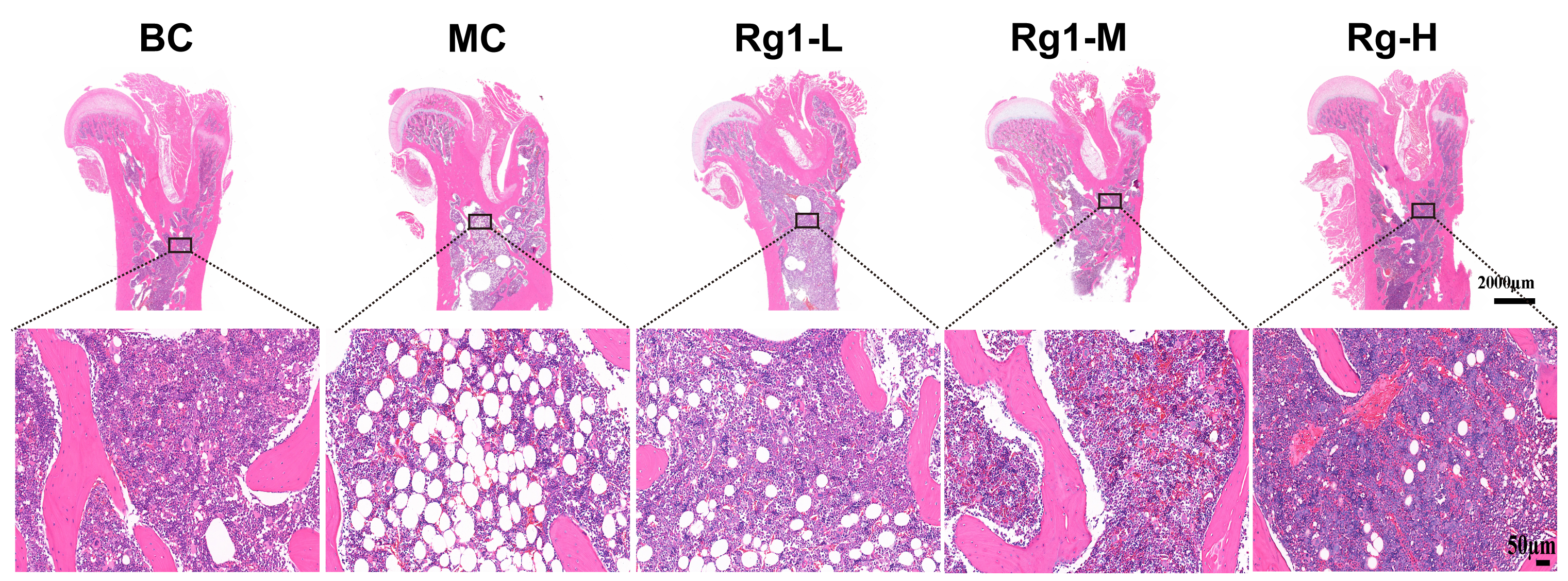

Supplement: Supplementary file 2 [file Image1.tif]
